# Spatial Tribological Properties of PI/PTFE Based Self-Stratifying Composite Coatings Grafted by Amino-POSS

**DOI:** 10.3390/polym18040521

**Published:** 2026-02-20

**Authors:** Chuanyong Yu, Min Wei, Qiwei Wang, Wei Zhang

**Affiliations:** 1 School of Mechatronic Engineering and Automation, Foshan University, Foshan 528225, China; yucy14@163.com (C.Y.); zhangwei18@fosu.edu.cn (W.Z.); 2 Institute of Advanced Wear & Corrosion Resistant and Functional Materials, Jinan University, Guangzhou 510632, China

**Keywords:** self-stratifying coatings, low earth orbit, high and low temperatures, atomic oxygen, friction and wear

## Abstract

In low Earth orbit (LEO), special environments such as atomic oxygen (AO), alternating high and low temperatures, and high vacuum can seriously affect the reliability and service lifetime of moving parts of space equipment. Therefore, there is an increasingly urgent demand for long-life, high-performance lubricating protective coatings with the rapid evolution of astronautical technology. In this study, polyimide (PI) was modified by polyhedral oligomeric silsesquioxane (POSS) with different numbers of functional groups to fabricate PI-based self-stratifying gradient composite lubricating coatings. The coating exhibited significantly enhanced AO resistance, and its vacuum tribological properties under alternating high and low temperature conditions were investigated. Results show that the mass loss of the gradient coating under AO exposure was significantly reduced by 78%, and the tribological properties of the coating under high and low temperature alternating conditions were significantly different. The friction coefficient was more stable and was smaller than that at high temperatures, and the wear rates of the POSS-modified coating also decreased by 77.5% and 50% for both high and low temperatures compared with that of the PI/PTFE coating.

## 1. Introduction

In low Earth orbit (LEO), harsh and extreme environments—such as atomic oxygen (AO), alternating high and low temperatures (−100~150 °C), high vacuum and ultraviolet radiation—have a serious impact on the polymer lubricating materials used in the spacecraft moving parts such as bearings and slides [1,2,3]. Especially for atomic oxygen, as it is one of the most significant components of the space environment, which can induce mass loss, surface roughening, and mechanical degradation in polymeric materials through oxidation etching [4,5]. Additionally, the high and low temperature cycling process is prone to generate internal stress between the coating and the substrate due to the mismatch of thermal expansion coefficients, potentially leading to cracking or peeling [6,7]. Meanwhile, the high-vacuum environment may promote the evaporation of traditional lubricants, thereby exacerbating friction and wear [8,9]. The coupling effects seriously threaten the reliability and operational lifetime of aerospace equipment during its in-orbit operation. Therefore, it is of great significance to develop lubrication materials with excellent AO; moreover, wear resistance has become an urgent demand for the sustainable development of aerospace technology.

To address the multiple performance requirements of the coating imposed by the complex space environment, the structural design concept of the gradient composite coatings has attracted extensive attention [10,11,12]. The gradient coating, through continuous variation of composition or structure, can achieve a harmonious integration of surface lubricity, intermediate mechanical support and strong substrate adhesion [13,14,15]. This integration effectively alleviates stress concentration and enhances interfacial bonding strength. Among these, self-stratifying gradient coatings utilize the spontaneous phase separation tendency of material components during the curing or film-forming process to form a layered structure with gradually changing chemical or physical properties in a single construction step [16,17]. This approach not only simplifies the preparation process but also improves the interlayer compatibility. Such coatings are particularly suitable for space applications, as they can optimize the distribution of lubrication and protective functions at the interface while enhancing the environmental stability and durability of the coating [18,19,20]. Lebeau et al. [19] prepared a self-stratifying epoxy/polyurethane-based coating using sulfur-containing compound as a curing agent, it exhibited excellent adhesion and mechanical properties in aeronautical applications. Zhao et al. [21] reported polyamide-imide-based self-stratifying coatings modified by polydimethylsiloxane, and the results revealed that the PDMS segments improved the AO resistance significantly. Thus, the self-stratifying gradient structure offers an efficient and feasible solution for space lubrication protection.

Polyhedral oligomeric silsesquioxane (POSS), as a nanoscale organic–inorganic hybrid materials, has a hollow cage-like framework composed of Si-O-Si bonds and surrounded by various organic functional groups, thereby endowing it with excellent compatibility and reactivity with polymers [22,23,24]. The incorporation of POSS can significantly improve the heat resistance, mechanical strength and AO resistance of polymer materials. On one hand, the inorganic Si-O-Si framework of POSS trends to form a dense, protective SiO_2_ passivation layer under high-energy AO irradiation, effectively inhibiting further oxidation erosion. On the other hand, its nanoscale effect enhances the intermolecular force between polymer chains, thereby improving the balance between coating hardness and toughness [25,26]. Moreover, by adjusting the type and numbers of functional groups, it is possible to precisely control the surface energy, phase separation behavior, and interface properties of the material [17,27]. POSS with multifunctional groups can be chemically bonded onto the polymer matrix acting as a cross-linking agent that is mainly distributed inside of the polyimide system, which improves the cross-linking density and interfacial strength of the resin. The mono-POSS were introduced as end-capping agent that is mainly distributed on the composite surface due to the surface migration, which resulted in the gradient structure. Yao et al. [28] reported a PI-based composite with excellent tribological properties and AO resistance was fabricated by employing MPA-POSS as a cross-linking agent. Kim et al. [29] employed reactive molecular dynamics to investigate the ablation resistance of POSS-PI nanocomposites, and the results suggest that POSS inhibits the exfoliation of large carbonic molecules and significantly contributes to the development of a ceramic passivation layer. Therefore, this tunability provides a key material basis for designing functional gradient coatings.

Based on the above perspective in mind, the polyimide (PI) was modified by incorporating mono- and octa-amino functionalized POSS. Leveraging the spontaneous phase separation behavior triggered by the difference in surface energy during the curing process, the PI-based self-stratifying gradient composite lubricating coatings were successfully constructed. The structural stability and mass loss of the composite coating after AO exposure were investigated, and the vacuum tribological properties under alternating temperature and humidity conditions were further studied. Through material design, structural characterization and performance research, the mechanism of the self-layer effect induced by POSS modification and the enhancement of environmental adaptability was clarified, providing experimental basis and theoretical reference for the aerospace lubrication protection coatings.

## 2. Materials and Methods

### 2.1. Materials

Mono- and Octa-amino polyhedral oligomeric siloxane (POSS) were obtained by Huawei Ruike Chemical Co. (HWRK, Beijing, China). 4,4′-Oxydianiline (ODA) was provided by Kefeng Chemical Reagent Co. (Shanghai, China). Pyromellitic dianhydride (PMDA) was purchased from Sinopharm Group Chemical Reagent Company (Shanghai, China). Polytetrafluoroethylene (PTFE) powders were purchased from the Shanghai Synthetic Resin Research Institute (Shanghai, China). N,N-dimethylacetamide (DMAc), hydrochloric acid and anhydrous ethanol were purchased commercially from Dongfang Instrument Chemical Glass Co., Ltd. (Shanghai, China). All the reactants were of analytical purity.

### 2.2. Preparation of the POSS-PI/PTFE Self-Stratifying Gradient Coating

**Figure 1:**
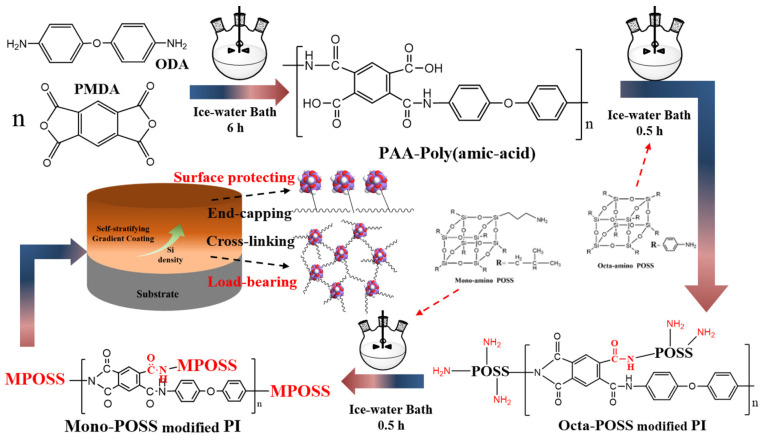
Schematic diagram of PI resin modified by mono- and octa-amino POSS.

An appropriate amount of ODA (1.5 g) was added to 30 mL of DMAc and stirred for 30 min. After the ODA powders had fully dissolved within the three-necked flask, the flask was placed in an ice-water bath. Then, the PMDA (1.65 g) powders were slowly added to the flask under continuous stirring in the ice bath. During this process, the viscosity of the reaction solution increased gradually, and the color changed from transparent to light yellow. Stirring was continued for another 6 h until the solution transformed into a polyimide precursor solution (PAA). Subsequently, octa-amino POSS (5 wt.%) and mono-amino POSS (5 wt.%) were separately added into the PAA mixture at 30 min intervals, followed by continuous stirring for 1 h to obtain the POSS-PI organic–inorganic hybrid resin. The preparation process is shown in Figure 1.

The POSS-PI composite resin was then blended with PTFE in a weight ratio of 50%. The mixture was then subjected to high-speed mechanical stirring followed by ultrasonic oscillation to achieve uniform mixing. Subsequently, the POSS-PI/PTFE composite slurries were sprayed onto the 316 L steel substrate. Before spraying, the substrate was sandblasted to remove the surface contaminants. The diamond sand with a particle size of approximately 25 μm was sprayed at a pressure of 0.8 MPa. The substrate was then ultrasonically cleaned in absolute ethanol for 10 min to eliminate any residual contaminants and abrasive particles, followed by drying in a drying cabinet at 60 °C for 20 min. The composite coatings were first dried for 1 h in an electric drying oven at 150 °C, and then cured at 280 °C for 1 h to obtain a lubrication and protection coating with a thickness of 25 ± 3 μm. After the coating were cured, these samples were stored in a constant-temperature drying cabinet at 25 °C to prevent external environmental effects on the coating surface.

### 2.3. Structure Characterizations

The functional group changes on the coating surface after atomic oxygen irradiation were measured using the Fourier Transform Infrared spectrometer (FTIR-ATR, Nexus 870, Thermo-Nicolet, Madison, WI, USA) in the spectral range of 500~4000 cm^−1^. The phase composition and elemental valence of the coating after AO exposure were investigated by employing the X-ray photoelectron spectroscopy (XPS; ESCALAB 250Xi, Oregon, OR, USA). The surface morphologies, cross-section and wear tracks were investigated using a scanning electron microscope (SEM, JSM-5600LV, JEOL, Tokyo, Japan) equipped with an energy dispersive spectrometer (EDS). The three-dimensional features, two-dimensional cross-sectional curves and surface roughness of the wear marks were measured using a three-dimensional topography measurement instrument (AEP, R-tec instruments, San Jose, CA, USA).

### 2.4. Atomic Oxygen (AO) Irradiation Experiments

The atomic oxygen (AO) exposure experiments were performed using a space environment ground simulation device (purchased from Yanfei Electrical Technology Company Limited, Hefei, China) under vacuum (<1.5 × 10^−2^ Pa). In these experiments, AO with an average kinetic energy of approximately 5 eV struck the lubricating coatings, and the AO flux was approximately 1.23 × 10^16^ AO/cm^2^·s. The AO erosion experiment was conducted 40 h, which was approximately equivalent to the exposure of a satellite surface to the LEO environment for 100 days [30].

The effective AO flux can be calculated by the following Equation:

$$AO\;flux=\frac{\Delta M}{\rho \times A\times t\times AORC}$$

where *ρ* is the density of Kapton, *A* is the exposed surface area, *t* is the exposure time, and *AORC* is the AO reactivity coefficient of Kapton with an AO reactivity of 3.0 × 10^−24^ cm^3^/atom. Additionally, Δ*M* is the mass loss of Kapton after AO irradiation [30].

### 2.5. Mechanical and Tribological Properties

The contact angles of the composite coatings were conducted by employing a contact angle measuring instrument, with the water droplet volume being 5 μL. The micro-hardnesses of these composite coatings were measured by a hardness tester (HXS-1000, Cai Kang, Shanghai, China) under a 10 g loading condition for a duration of 5 s. Under the same conditions, each sample coating was measured at least three times to obtain the final experimental result. The friction wear behavior in vacuum of the composite coating under both high and low temperature conditions was comparatively studied using a ball-disk type vacuum friction testing machine (CSM, Anton Paar, Shanghai, China). The vacuum pressure was less than 5 × 10^−5^ mbar. The diameters of the counterpart steel ball (GCr15, 58~62 HRC) were approximately 6 mm. The environmental temperature of the sample was raised from room temperature to 150 °C (from room temperature to −50 °C, both temperatures rise and drop times were 10 min). The dual balls moved at a speed of 10 cm/s (amplitude: 2.5 mm) on the coatings, with a load of 5 N and a friction distance of 150 m. Under the alternating high and low temperature conditions (−50~150 °C), the specific temperature changes over time are presented in the results and discussion section. The friction test was conducted at a speed of 10 cm/s (amplitude: 2.5 mm) under 5 N load, and the friction distance was 1050 m. The wear volumes were measured using the non-contact surface profilometer (Micro-XAM-3D, AEP, San Jose, CA, USA), and the wear rates were calculated to evaluate the tribological properties under high and low temperatures. All friction and wear experiments were repeated three times at least to ensure reproducibility. A specific wear rate (W) was calculated according to the following formula:

$$W=\frac{V}{F\times D}$$

where V, F, and D represent the wear volume (mm^3^), normal load (N), and wear distance (m), respectively.

## 3. Results

### 3.1. Morphologies and Physical Properties of the Composite

**Figure 2:**
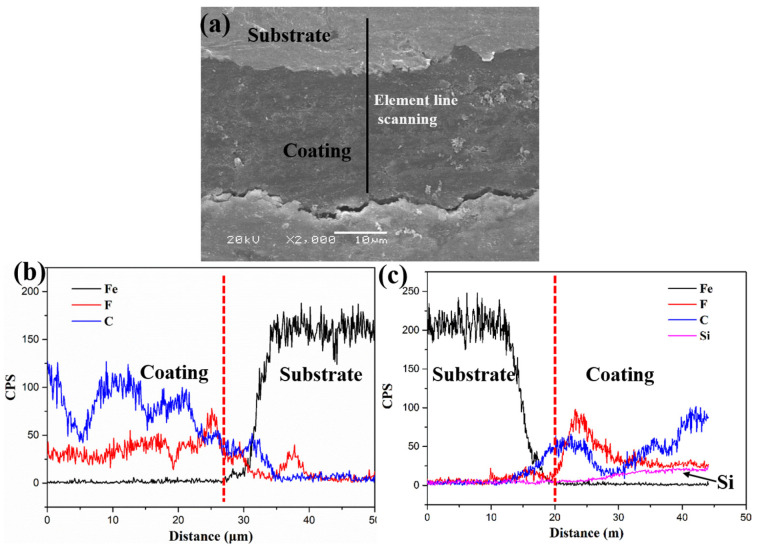
Cross-sectional SEM morphology (**a**) and element line scanning spectra of the (**b**) original and (**c**) POSS-PI/PTFE coating.

Figure 2 shows the cross-sectional SEM morphology of the composite coating, and the corresponding elemental line-scan spectra were investigated. As shown in Figure 2a, the lubricating coating is relatively uniform and adheres firmly to the substrate, with no gaps at the interface. The coatings thickness is approximately 25 ± 3 μm. In addition, the EDS liner profiles of the sectional coating were also investigated to confirm the distribution of the silicon (Si) element, as illustrated in Figure 2b,c. It can be seen that, the sectional morphology exhibits obvious interface between the substrate and coating according to the Fe element. There were no Si elements in the PI/PTFE composite coating, whereas the Si elements appeared clearly in POSS modified PI/PTFE coating. Furthermore, the Si content increased from inner to surface, which can be attributed to the surface migration effect and spontaneous phase separation of nano-particles. The gradient distribution of Si elements confirmed the formation of the self-stratifying gradient structure caused by the spontaneous phase separation during the coating formation process. Additionally, the result was confirmed by the atomic number percentage from coating surface to inner using the XPS etch, as shown in our previous work [17].

**Figure 3:**
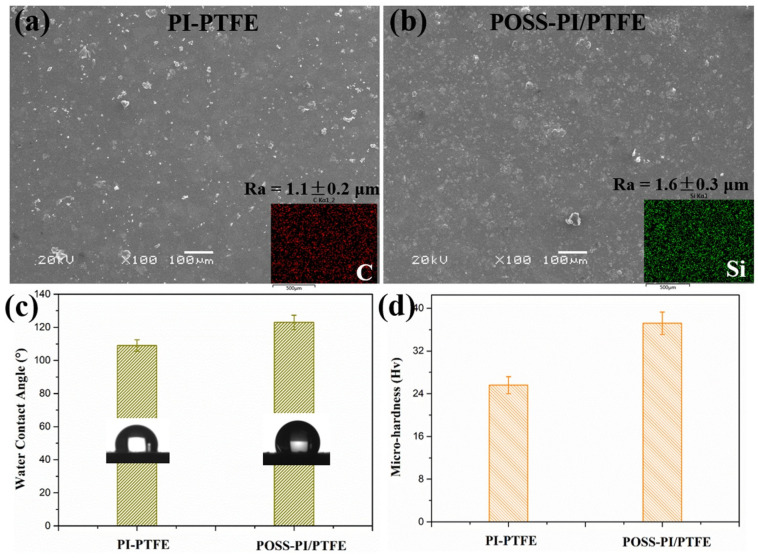
Surface morphologies (**a**,**b**), water contact angle (**c**) and hardness (**d**) of the original (**a**) and POSS-PI/PTFE (**b**) coatings.

Figure 3 exhibits the surface SEM morphologies and EDS element mapping of the composite coatings, as well as the water contact angle and surface hardness. It can be seen that the coatings surfaces are relatively smooth and continuous in Figure 3a,b, and the surface of the PI/PTFE coating exhibits a smaller roughness (1.1 ± 0.2 μm) compared with that of POSS-modified composite coating (1.6 ± 0.3 μm). This difference can be attributed to the surface migration effect and enrichment of POSS nanoparticles during coating formation, and the surface roughness supports this result [16,17]. Furthermore, the uniform distribution of carbon (C) and silicon (Si) elements indicates a homogeneous distribution of POSS within the composite material.

The water contact angle and microhardness of the original and POSS-modified PI/PTFE lubricating coatings were also studied (Figure 3c,d). As shown in Figure 3c, the surface enrichment of POSS contributes to a reduction in surface energy, resulting in an increase in the water contact angle. Additionally, compared with the PI/PTFE coating, the microhardness of the POSS-modified coating increased markedly. This enhancement may be caused by the introduction of the cage-like inorganic framework composed of Si-O-Si bonds in POSS, as well as the reaction between the octa-amino groups and the resin matrix, which collectively improved the load-bearing capacity of the coating.

### 3.2. Evolution of Morphologies, Microstructure After AO Exposure

**Figure 4:**
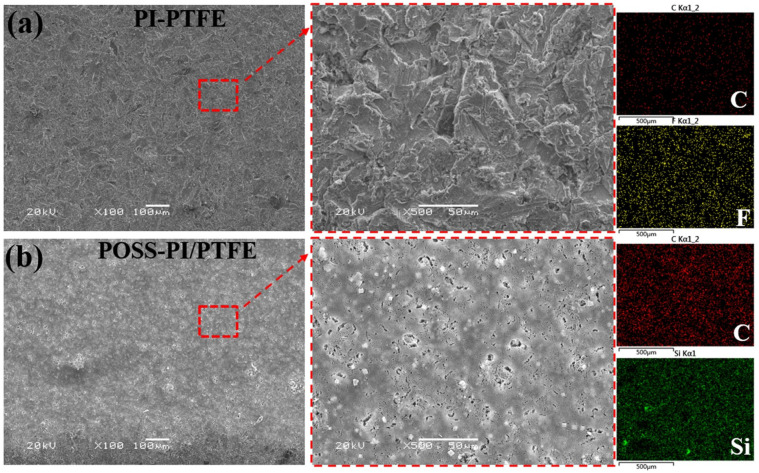
SEM micrographs and EDS spectra of the coating surfaces, (**a**) PI/PTFE, and (**b**) POSS-PI/PTFE coating.

The surface SEM micrographs and element distribution are shown in Figure 4 for both the PI/PTFE and POSS-PI/PTFE composite coatings after AO irradiation. Prior to AO irradiation, the coating surfaces as shown in Figure 3 were relatively smooth and continuous with low roughness. However, after AO exposure, the surface morphologies and micro-structure of the composite coating underwent significant damage. The surface of the PI/PTFE coating presents a rough blanket-like structure, which was completely damaged; whereas, the POSS-modified coating surface remains relatively smooth and dense, albeit with the presence of small micropores. Furthermore, compared with the unirradiated coating, it can be observed that the surface carbon (C) content of the PI/PTFE coating decreased markedly after AO irradiation (Figure 4a). This result indicates that the PI and PTFE on the coating surface have undergone severe oxidation and degradation, accompanied by the escape of volatile gases. It is worth noting that the POSS-modified PI/PTFE coating surface retains a relatively high C content, indicating that the smooth and dense oxide silicon passivation layer formed on the surface protects the PI and PTFE from further erosion and oxidation [31]. Additionally, after AO irradiation, the Si elements on the surface are uniformly and densely distributed for the POSS modified coating, with a significant increase in content. This can be attributed to the oxidation of POSS, which generates silicon dioxide and forms a dense protective layer on the coating surface.

**Figure 5:**
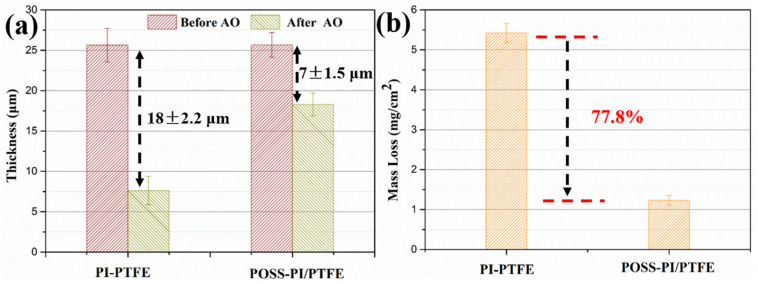
Thickness (**a**) and mass loss (**b**) of the composite coatings before and after AO irradiation.

The changes in thickness and mass loss per unit area of the composite coatings before and after AO exposure are shown in Figure 5. After AO irradiation, the thickness of the PI/PTFE coating decreased markedly by approximately 18 ± 2.2 μm, whereas the thickness was decreased by 7 ± 1.5 μm only for the POSS modified coating (Figure 5a). In addition, the mass of PI/PTFE coating decreased markedly after AO exposure, with a loss of approximately 5.43 mg/cm^2^. In contrast, the POSS-modified coating exhibited a lower mass loss at only 1.21 mg/cm^2^. Therefore, the reductions in mass loss and thickness can be attributed to the oxidation and degradation of the PI and PTFE on the coating surface, which generated a volatile constituent (CO and CO_2_) [19,21]. More importantly, the smaller reduction in thickness and mass loss of POSS modified coating was caused by the formation of a protective layer composed of silicon oxide.

**Figure 6:**
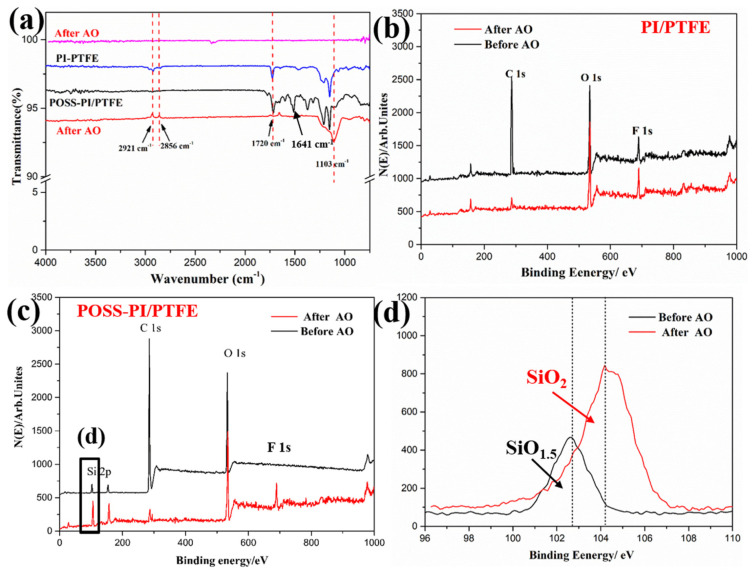
FTIR-ATR (**a**), XPS spectra of the (**b**) original and (**c**) POSS-PI/PTFE coating, and high-resolution Si 2p (**d**) spectra before and after AO irradiation.

Figure 6 shows the FTIR-ATR and XPS spectra of the PI/PTFE and POSS-PI/PTFE composite coatings before and after AO irradiation. It can be seen that, AO irradiation caused obvious variation in the intensity of the characteristic absorption peaks on the coating surface. After AO exposure, the peak intensities corresponding to 1641 cm^−1^ (C=O), 1720 cm^−1^ (C=C) and 2921 cm^−1^ (C-H) decreased significantly, indicating that there was oxidation and degradation of PI molecular chains on the coating surface (Figure 6a). These results indicate that some complex chemical reactions and degradation of PI molecules occurred following AO irradiation [32]. Similarly, the intensity of the peaks of the POSS-modified PI/PTFE coating also decreased obviously, which suggests that the organic PI and PTFE components on the coating surface still underwent severe oxidation degradation. However, a distinct characteristic Si–O peak appeared at 1103 cm^−1^, which revealed that the POSS nanomaterials were exposed on the coating surface and underwent oxidation under AO irradiation [30,33].

The XPS spectra were further employed to investigate the changes in the elements’ chemical composition and valence state of the PI/PTFE and POSS-modified composite coatings before and after AO exposure, as shown in Figure 6b–d. The original PI/PTFE coating exhibited XPS peaks corresponding to C1s, O1s, and F 1s; whereas an additional Si 2p peak was observed in the POSS-PI/PTFE coating. Upon AO irradiation, significant changes occurred in the atomic composition of the coating. In Particular, the C content decreased markedly, which is attributed to the oxidation and degradation of the organic components on the surface, leading to the release of volatile gases [3,21]. In contrast, the F content increased significantly because of the exposure of internal PTFE on the coating surface. Furthermore, the intensities of the Si 2p and O 1s peaks of the POSS-PI/PTFE coating increased obviously after AO irradiation (Figure 6c). This enhancement can be ascribed to the oxidation of POSS into non-volatile silica and related oxides, resulting in a significant rise in the content of oxygen and silicon elements after AO irradiation [25,29]. Finally, the high-resolution Si 2p spectra revealed a shift in binding energy from 102.6 eV to 104.1 eV, indicating a change in the silicon valence state from SiO1.5 to SiO2. This confirmed that POSS underwent oxidation and generated silicon dioxide upon AO exposure.

### 3.3. Tribological Properties Under Alternating High and Low Temperatures

In low Earth orbit (LEO), a spacecraft completes one full orbit around Earth in approximately 90 min, periodically passing through the direct sunlight zone and the Earth’s shadow zone, thus experiencing a complete cycle of high and low temperatures [34]. These temperature extremes significantly affect the performance of polymer materials. While the introduction of POSS has been shown to enhance the thermal stability and wear resistance of PI/PTFE based coatings, but the tribological properties under alternating high and low temperature cycling conditions remains to be further investigated.

**Figure 7:**
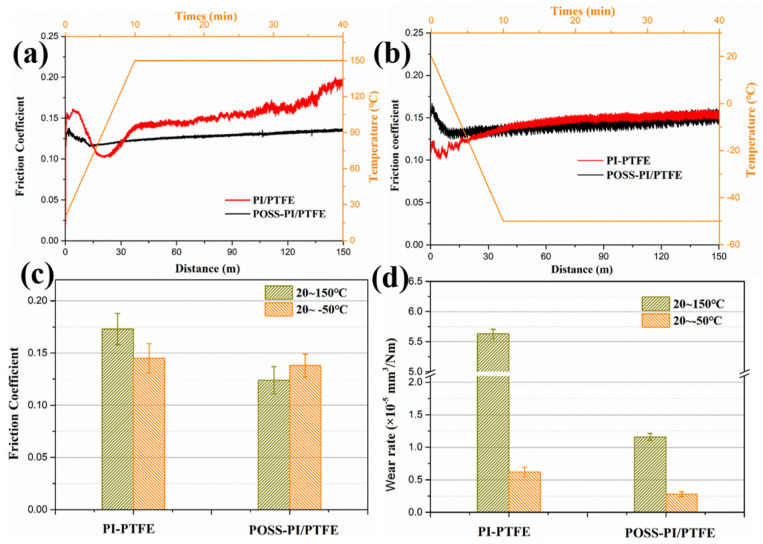
Friction coefficient curves (**a**,**b**), average CoF (**c**) and wear rates (**d**) of the original and POSS-PI/PTFE coatings under (**a**) high and (**b**) low temperatures.

Figure 7 presents the friction coefficient (CoF) curves and wear rates under both high and low temperature conditions as well as during the heating and cooling processes. It can be seen that, at a high temperature (150 °C), the CoF of the PI/PTFE composite coating initially decreased then increased during the heating process, fluctuating between 0.15 and 0.2. In contrast, the CoF of the POSS-PI/PTFE coating remained lower and more stable, around 0.13 (Figure 7a). For the PI/PTFE coating, the variation in the friction coefficient might be caused by the plastic deformation of the coating at high temperatures, which increases the contact area and consequently alters the frictional behavior. Meanwhile, the octa-amino groups and the hollow cage-like inorganic framework of POSS enhance the thermal stability and load-bearing capacity of the coating. At low temperatures (from room temperature to −50 °C), the friction coefficients of both the PI/PTFE and POSS-PI/PTFE coatings remain consistently low and relatively stable. Only minor differences were observed during the initial stage, and the friction coefficients under low temperatures are generally lower than those at high temperatures (Figure 7b). According to the CoF curves, high temperatures had a very marked impact on the original PI/PTFE coating. Additionally, for both the high and low temperatures, the friction coefficient of the POSS-modified coating is lower than that of the original coating, indicating that POSS has a positive impact on the tribological properties of the coating. These trends indicate that the lubrication performance of the PI/PTFE coating deteriorates at high temperature, whereas the POSS-PI/PTFE coating shows a smaller increase in the friction coefficient at high temperatures, suggesting improved heat resistance and better retention of lubricating properties.

Figure 7c presents the wear rates of the composite coatings under both high and low temperature conditions. At low temperatures, the wear rates of the original and POSS-modified coatings are significantly lower than those at high temperatures. The difference can be attributed to the softening and plastic deformation of the coatings at high temperatures. Moreover, under both high and low temperatures, the POSS-modified coatings exhibit a further reduction in wear rate. In summary, the incorporation of POSS into PI/PTFE coating improved the tribological properties significantly at high temperatures, helping to reduce the friction coefficient and suppressing wear, especially under high-temperature conditions [35].

**Figure 8:**
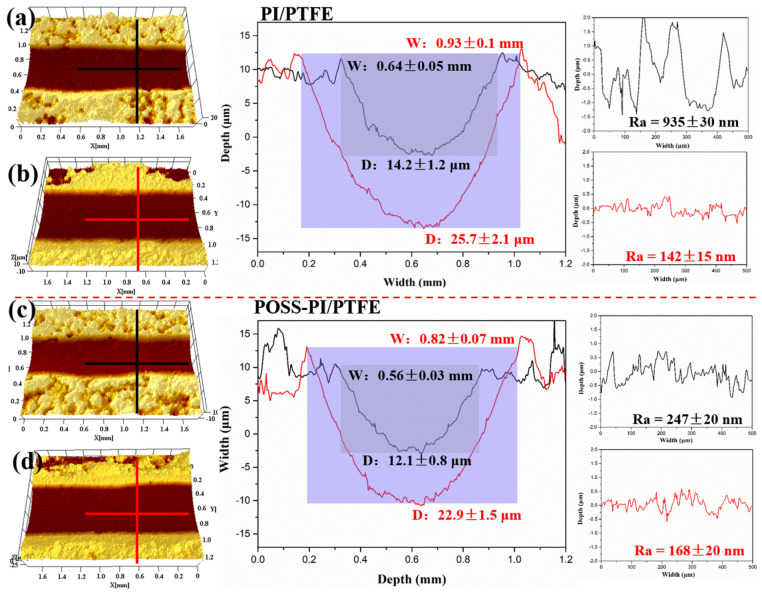
Three-dimensional (3D) morphologies and cross-sectional curves of original (**a**,**b**) and POSS-PI/PTFE (**c**,**d**) coatings under high (**b**,**d**) and low (**a**,**c**) temperatures; the black and the red line represent the cross-sectional curves of the wear tracks and the surface roughness of the coating under different conditions; Wand D represents the width and depth of the wear tracks, respectively.

The three-dimensional (3D) morphologies and corresponding cross-sectional curves of the wear tracks under high and low temperature conditions were studied to further investigate the wear mechanism, as shown in Figure 8. Under low temperatures (−50 °C), compared with the width and depth of the original coating (0.64 mm and 14.2 μm in Figure 8a), the wear track of the POSS-modified coating was relatively narrow and shallow (0.56 mm and 12.1 μm in Figure 8c). Correspondingly, the worn surface of the original coating showed a roughness (Ra) of 935 ± 30 nm, whereas that of the POSS-PI/PTFE composite coating was only 247 ± 20 nm. Under high-temperature condition (150 °C), the width and depth of the wear tracks significantly decreased from 0.93 mm and 14.2 μm to 0.82 mm and 22.9 μm. This result is primarily caused by the severe plastic deformation of the PI matrix at high temperatures, which served as the supporting materials in the composite coating, resulting in overall softening of the material. Notably, the worn surface inside the track exhibits a relatively low roughness of about 150 nm, which can be attributed to the smoothing effect caused by the softened coating during wear. It is worth noting that, under both high and low temperatures, the wear tracks of the POSS-modified coating are relatively smaller compared with the original PI/PTFE coating. The enhancement stems from the introduction of POSS, which improved the prominent cross-linking density and surface hardness, thereby significantly enhancing its wear resistance.

**Figure 9:**
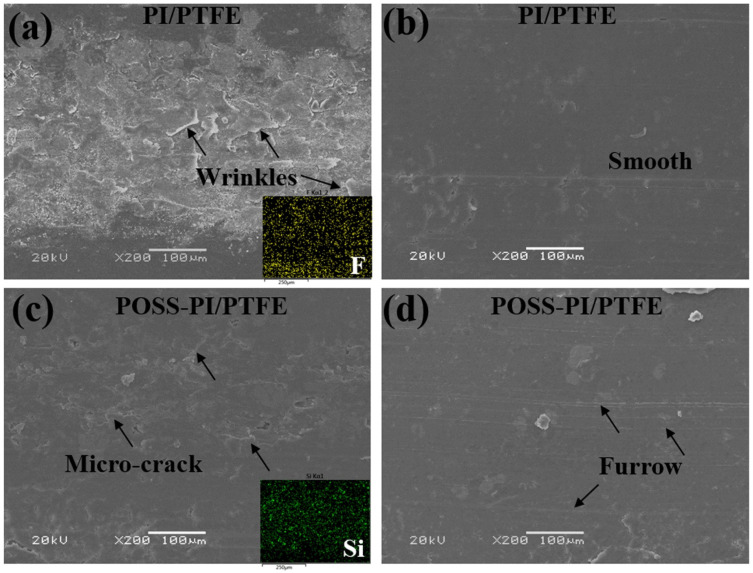
SEM morphologies of the wear tracks on the composite coatings under low (**a**,**c**) and high (**b**,**d**) temperatures.

The SEM morphologies and corresponding element distribution of the wear tracks under high and low temperatures are shown in Figure 9. At low temperatures (Figure 9a), the worn surface of the unmodified coating exhibits slightly curled wrinkles, resulting in a relatively rough morphology consistent with the 3D morphology (Figure 8a). In contrast, under high temperatures, the wear track appears smoother with lower roughness (Figure 9b).

For the POSS-modified coating at low temperatures, the worn surface shows only a few micro-cracks, and remains relatively smooth. While at high temperatures, the worn surface became even smoother but displays shallow furrows, likely caused by the presence of hard POSS particles. Furthermore, the F and Si elements are evenly distributed on the worn surface, which can provide excellent lubrication and load-bearing capabilities. Additionally, these SEM morphologies align well with the 3D morphologies results presented above.

**Figure 10:**
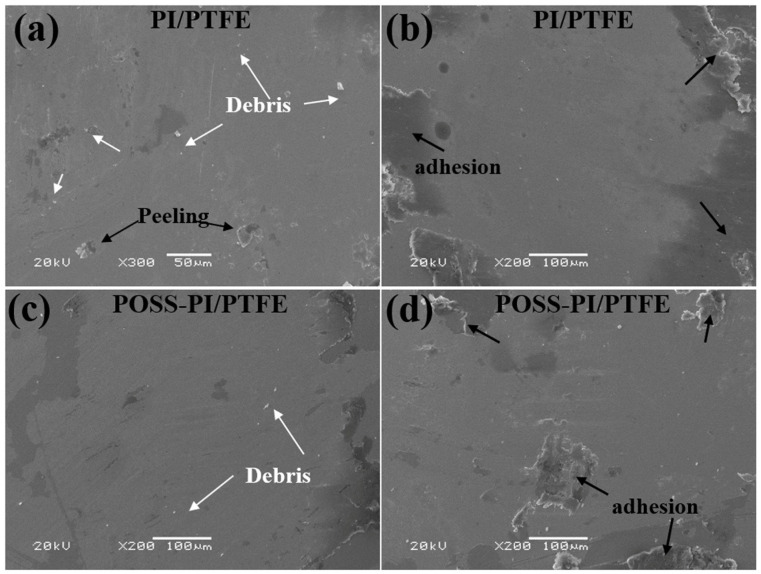
SEM morphologies of the worn surfaces on the counterpart balls under (**b**,**d**) high and (**a**,**c**) low temperatures for the composite coatings.

The SEM morphologies of wear areas on the corresponding steel balls were also explored, and the result are shown in Figure 10. The morphologies characterization of the worn surface reveals the formation of smooth and continuous transfer films within the wear areas. At low temperatures, a little abrasive debris appeared on the worn surface, along with minor adhesive traces around the wear zones. However, under high-temperature conditions, both the original and POSS-modified coatings showed more pronounced adhesion around the wear spots. This increased adhesion is attributed to the softening of the coatings at elevated temperatures, which leads to severe plastic deformation.

**Figure 11:**
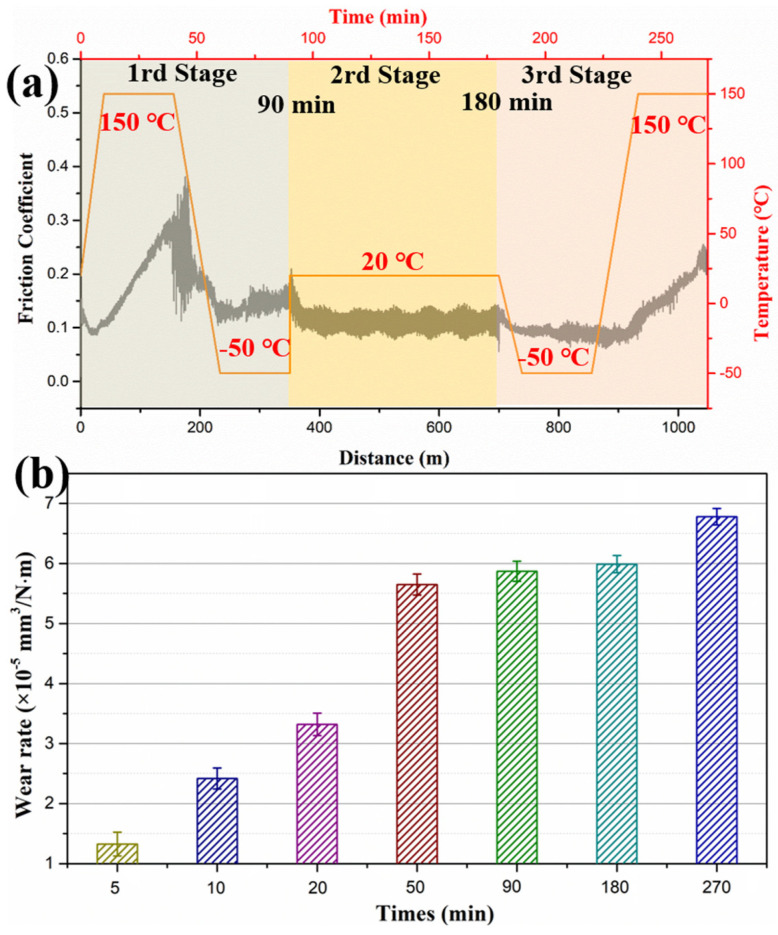
Friction coefficient (**a**) and wear rates (**b**) of the POSS-PI/PTFE coating under high and low temperature alternating cycling conditions.

High and low temperatures exerted distinct effects on the tribological properties of the composite coatings. Generally, the wear rates observed at low temperatures are lower than those at high temperatures, which is caused by the plastic deformation of the coating under thermal stress. To simulate actual space environment, the friction and wear tests were conducted under alternating cyclic high–low temperature conditions to investigate the tribological properties, and the results were divided into three stages as shown in Figure 11. As the temperature changed, the friction coefficient (CoF) of the coating rose sharply during the heating process, and continued to increase after reaching 150 °C. During cooling to −50 °C, the CoF also decreased and showed significant fluctuations. When the temperature stabilized at −50 °C, the CoF tends to stabilize at approximately 0.15. Upon reheating to 20 °C in the second stage, the CoF slightly decreased to around 0.13. In the third stage as the temperature again drops to −50 °C, the coating CoF remained relatively stable. However, during the subsequent heating process, the CoF increased accordingly (Figure 11a).

The friction experiment was divided into three stages corresponding to the temperature cycles. The wear rates at different stages were measured to study its tribological properties (Figure 11b). During the initial 20 min under high temperatures, the wear rate increased sharply, primarily caused by the softening of the coating materials, which induced severe plastic deformation and accelerated wear. When the temperature dropped to −50 °C (at 50 min), the wear rate remained stable throughout both low-temperature and subsequent room temperature periods. Upon reheating to 150 °C, the wear rate slightly increased. These results indicate that a high temperature has a more pronounced adverse effect on the wear resistance of the coating, whereas at a low temperature, the coating hardens and the wear rate remains essentially constant.

**Figure 12:**
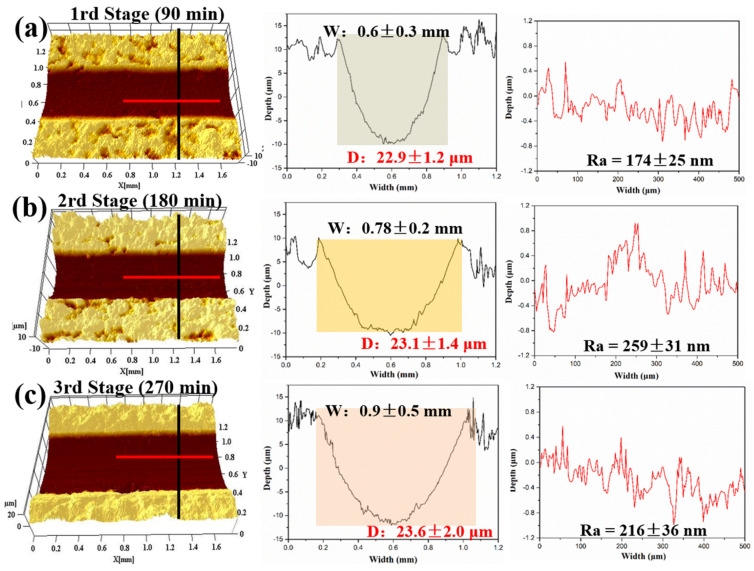
3D morphologies and cross-sectional curves of wear tracks under high and low temperature alternating conditions of POSS-PI/PTFE coating; Wand D represents the width and depth of the wear tracks, respectively.

The 3D morphologies and cross-sectional curves of the wear tracks were studied to further investigate their wear mechanism, as shown in Figure 12. In the first stage, the POSS-PI/PTFE coating suffered severe wear, with deep wear track (0.6 mm and 22.9 μm in Figure 12a). In the second stage, both the width and depth of the wear track slightly increased at room temperature (0.78 mm and 23.1 μm in Figure 12b). In the third stage, the width and depth showed a more pronounced increase (0.9 mm and 23.6 μm in Figure 12c). In addition to these results, the roughness of the worn surfaces in the three stages were measured as 174, 259 and 216 nm, respectively, which was in line with the friction results of the coating under high temperature and normal temperature conditions.

**Figure 13:**
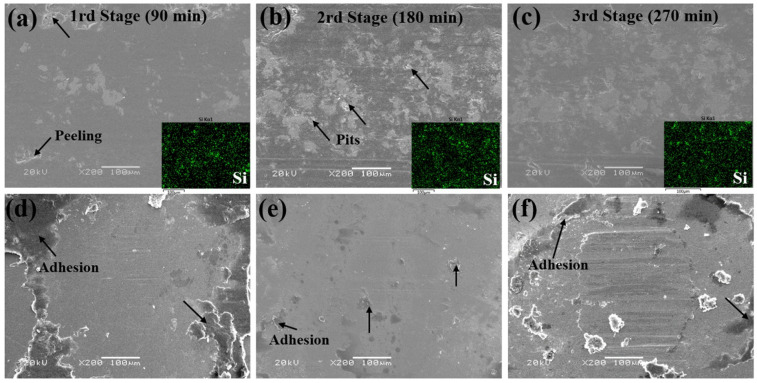
SEM morphologies and element distribution of wear tracks of POSS-PI/PTFE coating and counterpart balls under high and low temperature alternating conditions.

The SEM morphologies of the wear tracks of the composite coatings and counterpart balls are shown in Figure 13. After the first stage, the worn surface was relatively smooth, with slight peeling observed at the edges. Following the second stage, the worn surface became rougher and exhibited slight pits. After the third stage, the worn surface became relatively flat and smooth after undergoing a low-high temperature cycle. The worn surfaces after the first and third stages were relatively smooth, which is mainly caused by the softening and plastic deformation of the coating under high temperatures. The worn surfaces of the counterpart balls also verified this result. After the first and third stages, a larger amount of coating material was adhered around the dual ball worn areas, which was caused by the high and low temperature cycles. In contrast, after the second stage, there was significantly less adhesion on the worn areas at room temperature. It is important that after all three stages, uniform and dense transfer films predominantly composed of PTFE and PI were formed at the worn surface of the dual balls, thereby providing excellent lubrication for friction [36].

## 4. Conclusions

In view of the severe challenges posed by the harsh conditions such as atomic oxygen and temperature cycling on polymeric lubricating coatings in the LEO environment, a self-stratifying gradient composite coating with outstanding AO resistance and tribological properties was fabricated by covalently grafted mono- and octa-amino POSS with a unique inorganic cage-like framework onto the PI matrix. After AO exposure, the POSS nanomaterials underwent oxidation and formed a dense silicon oxide passivating protective layer on the coating surface, the thickness and mass loss of the coating significantly decreased by 61% and 78%, respectively. Furthermore, the incorporation of POSS significantly enhanced the hardness and wear resistance of the composite lubricating coating under the high and low temperature conditions. The wear rates under high and low temperatures were reduced by 77.5% and 50%, respectively. During the friction experiments conducted under the alternating high and low temperature conditions, the wear rate of the POSS-PI/PTFE coating remained relatively stable, demonstrating excellent tribological properties. Therefore, the POSS-PI/PTFE gradient coating exhibited superior AO resistance and tribological properties under current high and low temperature conditions in this work, showing considerable potential for application in spacecraft operating in low Earth orbit. Additionally, the further durability testing under extended high-low temperature cycling would be required for engineering adoption.

## Data Availability

The original contributions presented in this study are included in the article. Further inquiries can be directed to the corresponding authors.
